# Acute psychotic symptoms following a single dose of levofloxacin

**DOI:** 10.1002/ccr3.1240

**Published:** 2017-11-16

**Authors:** Larissa Takser, Roland Grad

**Affiliations:** ^1^ Department of Pediatrics Université de Sherbrooke Sherbrooke Quebec Canada; ^2^ Herzl Family Practice Centre Department of Family Medicine McGill University Montreal Quebec Canada

**Keywords:** Brain, levofloxacin, psychosis, side effects

## Abstract

The objective of our case report was to raise awareness of the neurotoxic potential of levofloxacin in naïve patients. In patients with severe infections and comorbidities, it would be difficult to discriminate the neurotoxic effects of levofloxacin from those of the medical condition itself. Thus, health professionals should be aware of the early onset CNS effects of levofloxacin in naïve patients.

## Introduction

In 2016, the FDA stated that considering the side effects of fluoroquinolones, they “should be reserved for those [patients] who do not have alternative treatment options” [https://www.fda.gov/Drugs/DrugSafety/ucm511530.htm]. However, the tolerance profile of levofloxacin is considered to be better than most, if not all of the fluoroquinolones available 1. We report a case of a young, previously mentally healthy woman with psychotic features after a single therapeutic dose of levofloxacin.

## Case Report

A 22‐year‐old woman was brought by her grandfather to our family medicine clinic several days following an acute onset of a sense of derealization, depersonalization, agitation, auditory hallucinations, insomnia, and nightmares. The patient and her grandfather confirm that she was always oriented in time and space as well as being self‐aware. The symptoms began at home, three hours after taking a single dose of levofloxacin 500 mg per os for a presumed urinary tract infection. She continued to experience symptoms for 3 days after which they completely resolved. The patient was not a user of alcohol or tobacco nor of illicit drugs. Her maternal history is negative for mental or neurological disorders; however, the patient's biological parents separated when she was a child. She reports that her father was homeless immediately after the separation and was a cocaine user.

## Discussion

Levofloxacin is rapidly absorbed after oral administration and within 60 minutes reaches plasma concentrations identical to those obtained from intravenous administration of the same dose [https://www.accessdata.fda.gov/drugsatfda_docs/label/2016/020634s067,020635s073,021721s034lbl.pdf]. In addition to a bactericidal effect due to inhibition of DNA gyrase, levofloxacin has been shown to inhibit GABA receptors 2. It has been shown that individuals at risk of and those with a first psychotic episode are deficient in GABA‐dependent inhibitory cortical control 3, 4. Thus, the features in our patient may be related to the collateral pharmacological effect of levofloxacin on GABA receptors.

The evidence on the induction of psychotic symptoms by levofloxacin is limited. There are two reported cases of levofloxacin‐induced psychosis in patients with significant comorbidities 5, 6. A case of delirium with psychotic features was reported in a previously healthy 42‐year‐old woman, but only after four days of treatment in combination with other medications 7. In a retrospective study of 73 patients treated for *H. pylori* 8 who developed acute neuropsychiatric symptoms, one case of insomnia associated with levofloxacin was reported.

## Conclusion

The objective of our case report was to raise awareness of the neurotoxic potential of levofloxacin in naïve patients. Fortunately, our patient was exceptionally perspicacious and insightful, and thus suspected a link between the medication and the onset of symptoms. This helped to elucidate the potential cause of her symptoms. In patients with severe infections and comorbidities, it would be difficult to discriminate the neurotoxic effects of levofloxacin from those of the medical condition itself. However, in our case, this adverse effect is probably independent of the underlying infection. Thus, health professionals should be aware of the previously unreported early onset CNS effects of levofloxacin in naïve patients.

## Authorship

LT and RG: contributed equally to the development of the idea, the writing, and critical revision of the manuscript. RG: collected relevant clinical data and obtained informed consent from the patient.

## Conflicts of Interest

Drs Takser and Grad report no competing interests.
